# Supplementary figures and images for: Genetic deciphering of the antagonistic activities of the melanin-concentrating hormone and melanocortin pathways in skin pigmentation
Source: PLoS Genet. 2020 Dec 10;16(12):e1009244. doi: 10.1371/journal.pgen.1009244 (PMC7755275; doi:10.1371/journal.pgen.1009244)

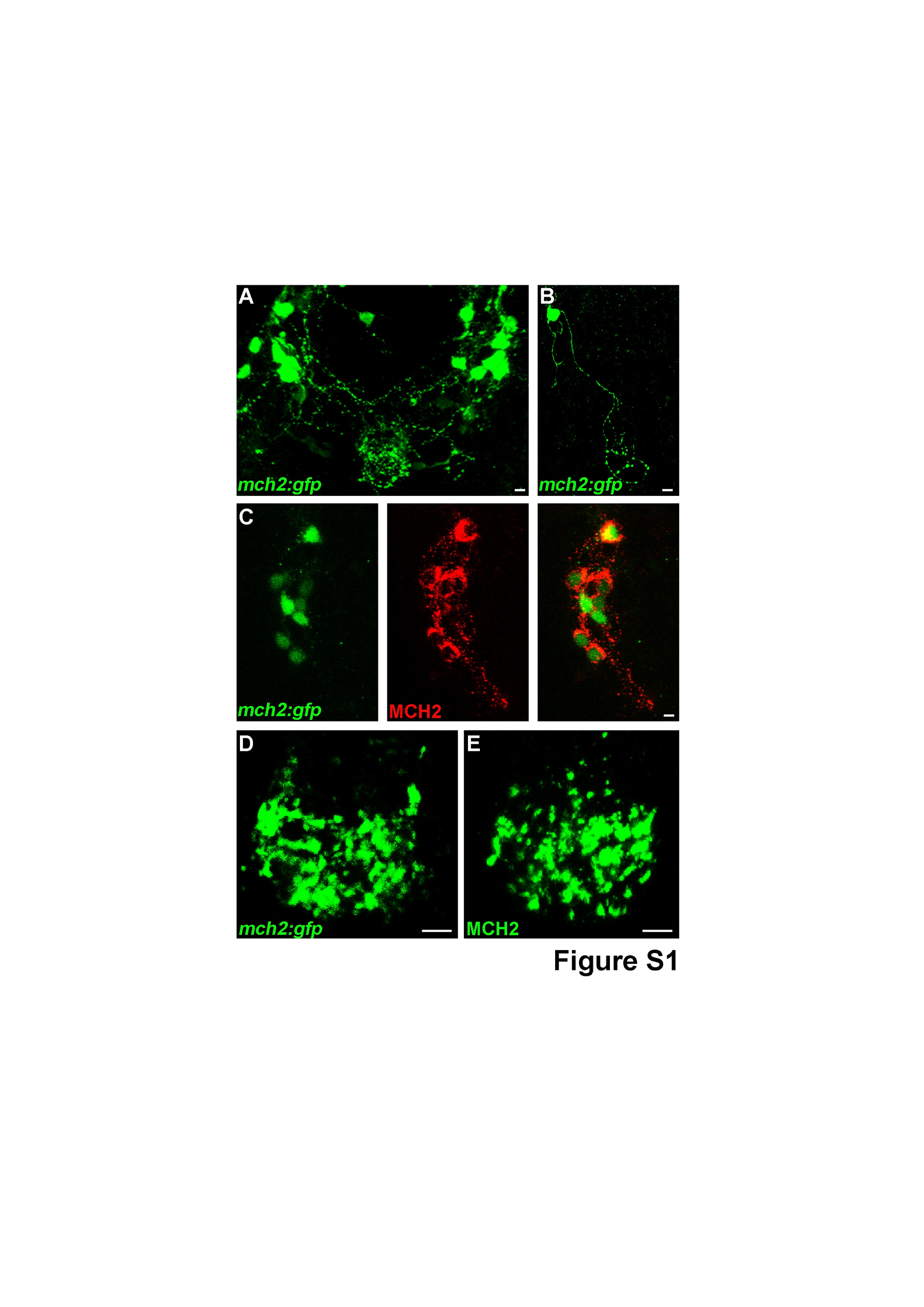

Supplement: S1 Fig — (A, B) Confocal projection of immunolabelling with EGFP in Tg(mch2:egfp) hypothalamus at 5 (A) or 3 (B) dpf, reveals that MCH2 neurons in the hypothalamus have axonal projections on the pituitary gland. (C) Confocal section of double immunolabelling showing overlap in the expression of endogenous MCH2 peptides and EGFP in Tg(mch2:egfp) at 5 dpf. (D, E) Confocal projection of immunolabelling with EGFP in Tg(mch2:egfp) (D) or with MCH2 (E) at 7 dpf, showing identical projection patterning of MCH2 neurons on the neurohypophysis with both tools. Ventral view with anterior up. Scale bars: 10 μm. (TIF) [file pgen.1009244.s001.tif]

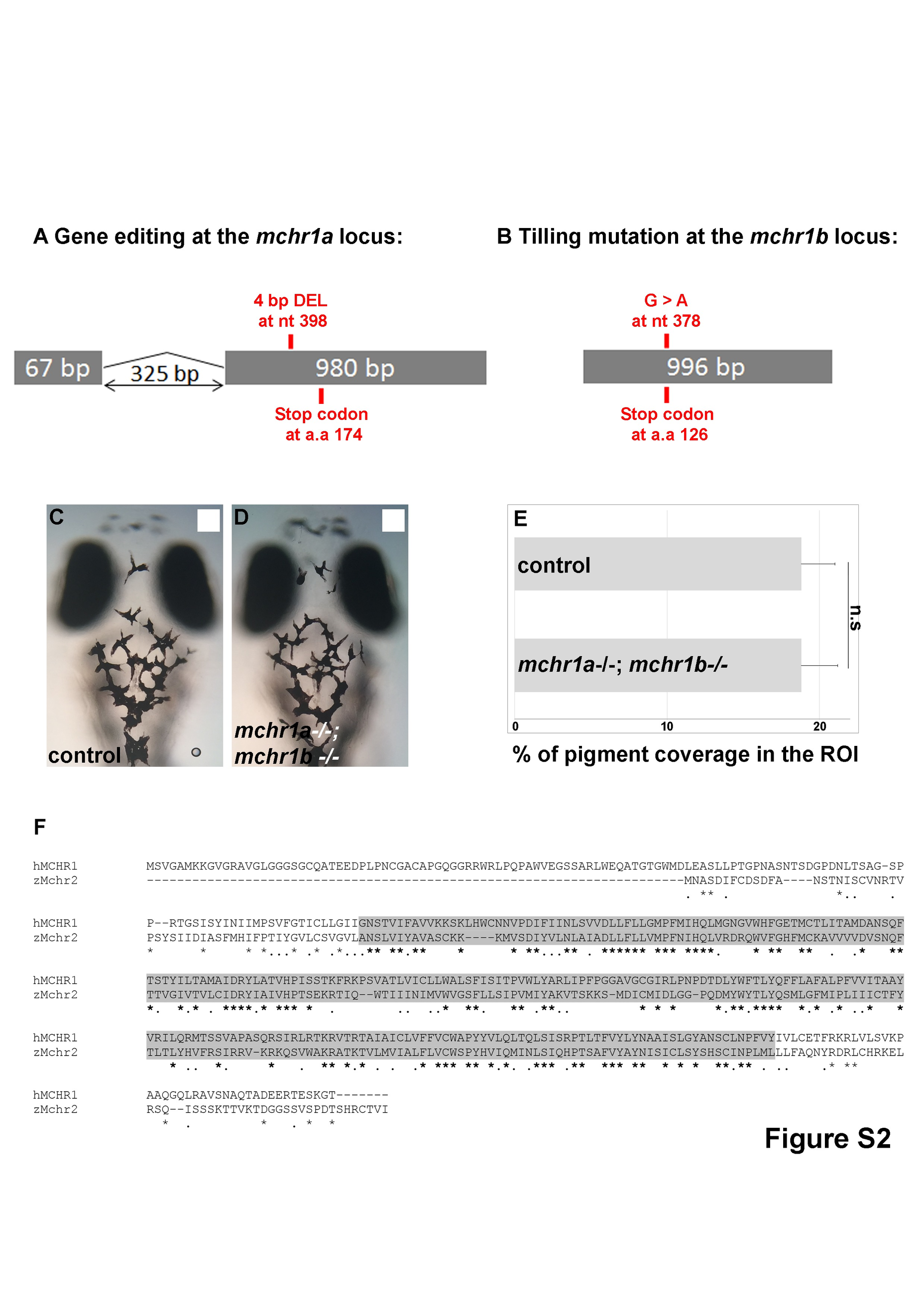

Supplement: S2 Fig — (A) Schematic representation of the mchr1a locus and the small genomic deletion induced by CRISPR/Cas-9 leading to a premature stop codon in the mchr1a coding sequence. (B) Schematic representation of the mchr1b locus and the nucleotide substitution leading to a premature stop codon in the mchr1b coding sequence. (C, D) Dorsal melanocytes of control (C) or mchr1a; mchr1b double homozygous mutant (D) white adapted larvae at 7 dpf. (E) Melanosome coverage was quantified in control or mchr1a; mchr1b double homozygous mutant white adapted larvae at 7 dpf. Dorsal view with anterior up. (F) Amino acids sequence alignment between the zebrafish Mchr2 and the human MCHR1 proteins. The highlighted sequence shows the conservation of the G-protein receptor domain. Dorsal view with anterior up. Error bars represent s.d. *P<0.05, **P<0.001, ***P<0.0005, determined by t-test, two-tailed. (TIF) [file pgen.1009244.s002.tif]

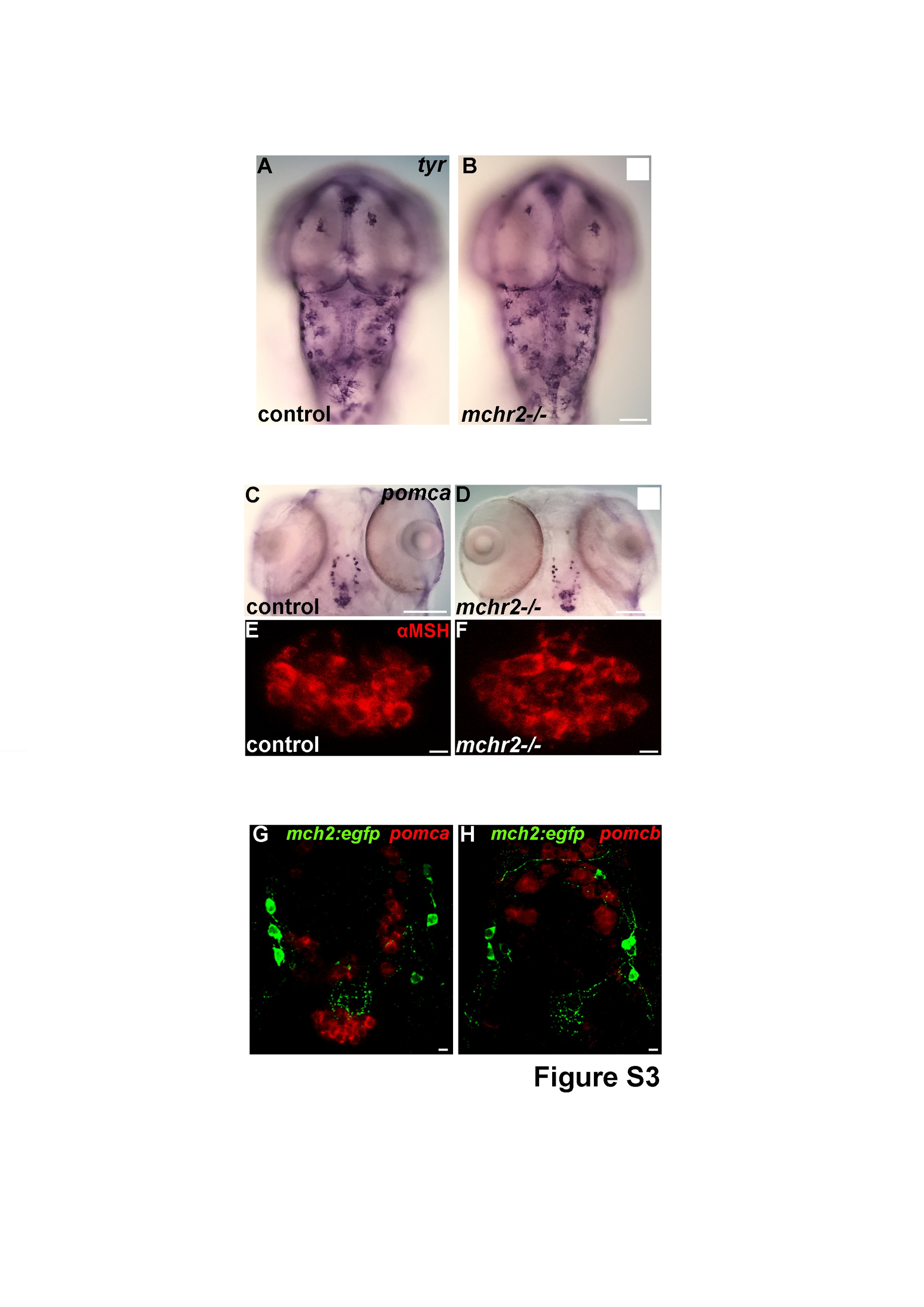

Supplement: S3 Fig — (A, B) Whole-mount in situ hybridization against tyrosinase (tyr) in larvae at 3 dpf, showing that melanocyte organization appears to be normal in the mchr2 homozygous mutant. (C, D) Whole-mount in situ hybridization against pomca in larvae at 5 dpf, showing that pomca expression appears to be normal in the mchr2 homozygous mutant. (E, F) Confocal projection of immunolabelling with α-MSH, showing that α-MSH expression appears to be normal in the mchr2 homozygous mutant. (G, H) Confocal section of double in situ/immunolabelling with pomca (G) or pomcb (H) in Tg(mch2:egfp) at 5 dpf, showing that only pomca is expressed in the pituitary gland. Dorsal view with anterior up (A, B). Ventral view with anterior up (C-F, I and J). Scale bars: 100 μm (A-D) or 10 μm (E, F, I and G). (TIF) [file pgen.1009244.s003.tif]

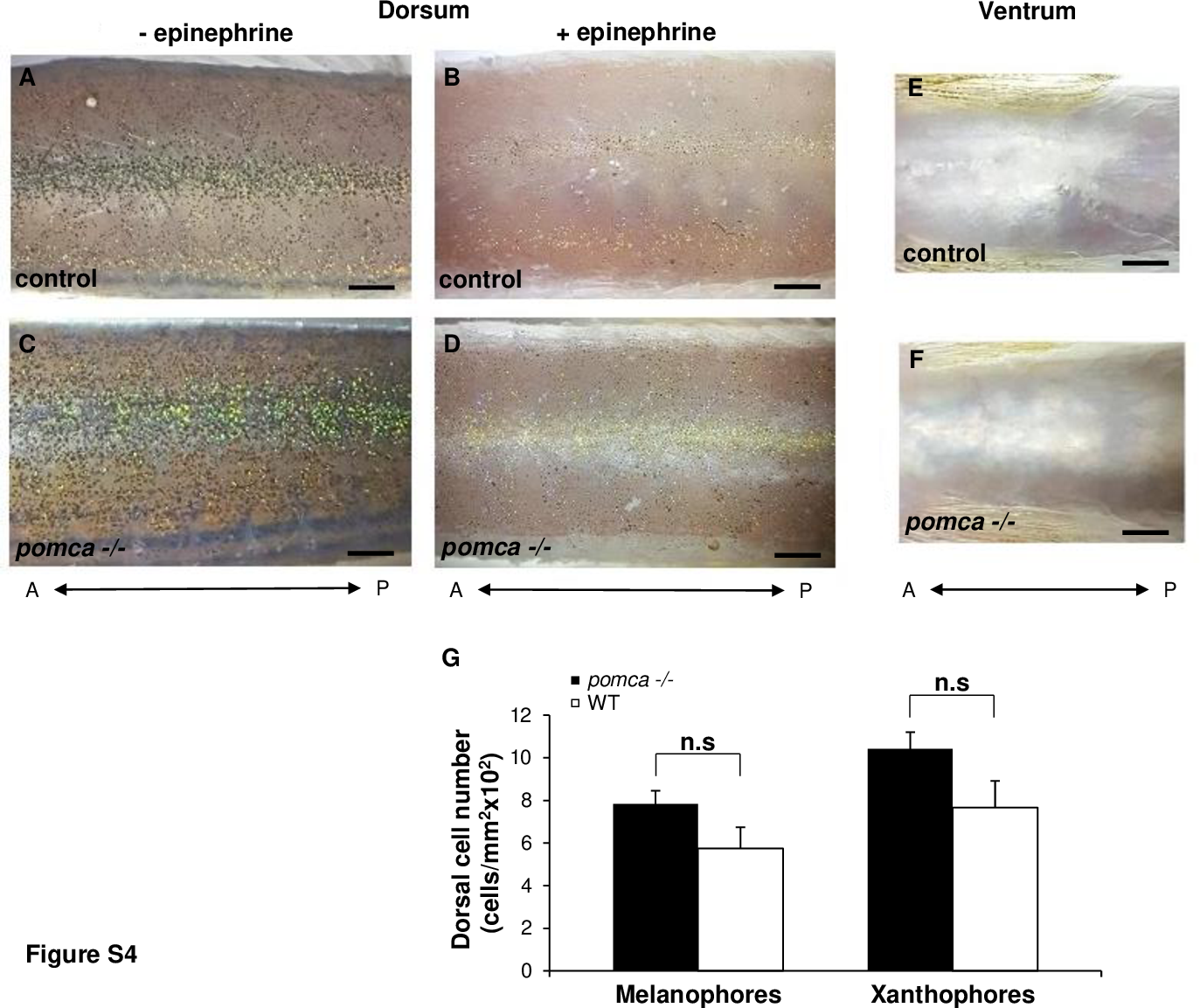

Supplement: S4 Fig — (A-D) Dark dorsums of 3-year-old pomca-/- and WT control sibling fish before (A, C) and after (B, D) epinephrine treatment to contract melanosomes. (E, F) Light ventrums of adult control (E) and pomca-/- (F) fish. (G) Quantification of dorsal melanophore and xanthophore counts of adult pomca-/- and WT control fish in 1mm2 regions after epinephrine treatment. Difference in each category is statistically insignificant (n.s; p-value = 0.07). Data shown as mean ±standard error of the mean (SEM). Axis denote anterior (A) to posterior (P) positioning of the fish. Scale bar: 1 mm (A-F). (TIF) [file pgen.1009244.s004.tif]
