# Supplementary material for: Genetic deciphering of the antagonistic activities of the melanin-concentrating hormone and melanocortin pathways in skin pigmentation
Source: PLoS Genet. 2020 Dec 10;16(12):e1009244. doi: 10.1371/journal.pgen.1009244 (PMC7755275; doi:10.1371/journal.pgen.1009244)
Supplement: Attachment — Submitted filename: Response to All Reviewers 20201012.pdf [file pgen.1009244.s010.pdf]

Response to All Reviewers:

Reviewer #1 (Comments to the Authors/Response to the Reviewer):

In the manuscript entitled 'Genetic deciphering of the antagonistic activities of the melanin-concentrating hormone and melanocortin pathways in skin pigmentation' Madelaine et al. show that background adaptation in zebrafish, i.e. aggregation or dispersal of melanosomes within melanophores, is regulated by two opposing signaling systems. The authors show that projections from melanin-concentrating hormone (MCH) producing neurons are found in close proximity to blood vessels in the pituitary, suggesting that this is the way the peptides are released to exert their functions in the periphery. Overexpression of the two MCH orthologs induces the aggregation of melanosomes in larval zebrafish, and the authors show that the peptide hormones act specifically via one of the receptors, Mchr2. Mutations in the gene encoding this receptor affect background adaptation not only in larvae but also in adult fish. In addition they show that the opposite process, i.e. melanosome dispersal, is defective in larvae with mutations in the pomca gene, which presumably affects melanocortin signaling via the receptor Mc1r. Surprisingly, while mchr2-/- is epistatic over pomca-/- in larvae, this is no longer the case in adult fish, which hints at a more complex regulation of the process.

The manuscript is generally well written and easy to understand; I only think that the introduction and the discussion could be a little bit more concise. The results are presented in a clear fashion and the conclusions are sound.

We are extremely grateful to the reviewer for recognizing the appeal of the results and the strength of our conclusions. As suggested we have shortened introduction and conclusion. We removed redundant elements present in the discussion such as references to human skin disorders as well as the broader impact of fish biology in pigmentation and oncogenic processes. While originally over 1,100 words, the introduction is now 750 words long.
The original Discussion section has also been shortened accordingly, but it now also contains information requested by Reviewer 3 regarding the potential involvement of receptor dimerization.

The only point, which I feel should be clarified concerns the pomca mutants generated and the relation to Mc1r signaling:
- the authors describe that two small deletions in different exons were produced, but they fail to indicate which allele (or combination) the animals shown in figures 4 and 5 actually carry.
- are the phenotypes of animals homozygous for either mutant allele identical?

We apologize for the confusion, the mutant allele actually contains both the deletions. The *pomca-/-* animals are homozygous for this single allele.
We have now clarified this point in the text. Pages 15 (results) and 25 (methods). It now reads "The mutant allele carries two deletions generated by CRISPR/Cas9 editing (one in each coding exon of the pomca ORF; Fig. 5A)"
Please find actual sequence below:

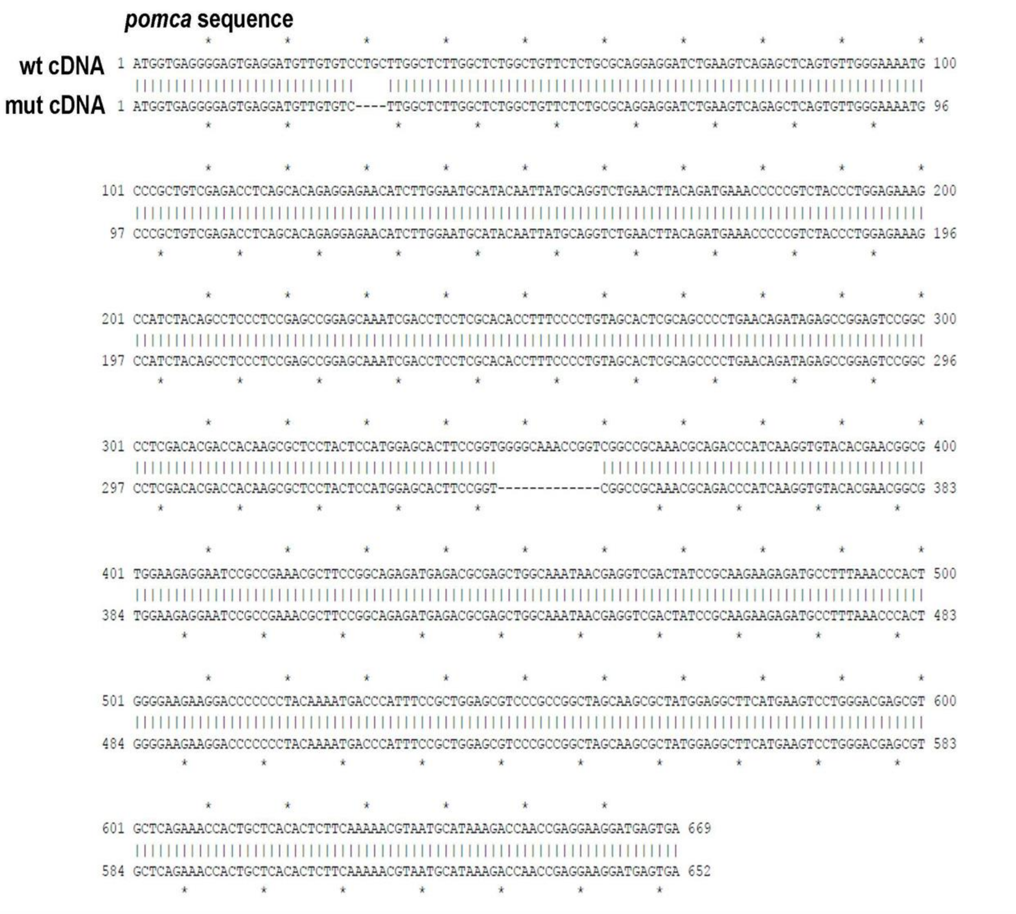

- how does the pomca k.o. phenotype compare to the published mc1r k.o. phenotype?
- especially, what is the number and distribution of pigment cells in the adult mutant fish? Is it similar to wild type, meaning only background adaptation in the mutant larvae is affected and there is no adult phenotype? Or, is it similar to mc1r mutants, which have not been described to be defective in background adaptation, but rather in counter shading? This could possibly provide further clues to understanding the complexity of the system.

Thank you very much for this interesting point. We referred to the publication, "*Loss-of-function mutations in the melanocortin 1 receptor cause disruption of dorso-ventral countershading in teleost fish*" from Cal and colleagues, 2019 (PMID: 31251842) for comparison as we do not host the mc1r mutant in our facility. While mc1r k.o. have hyperpigmented yellow belly, pomca k.o. retain a silver white coloring similar to wildtype. Overall, pomca k.o. has a relatively normal appearance and is hard to distinguish from wildtype and indeed is only affected in background adaptation.
To further characterize the pomca mutant we performed epinephrine treatment to contract melanosomes as described in Cal et al., 2019. As presented in the new figure S4, even though pomca k.o. dorsum seems slightly darker, precise quantification and statistical analysis reveal no significant difference with control. Also, as mentioned above, Fig. S4 displays similar ventrum in pomca k.o. and wildtype control siblings. The absence of marked pigmentation phenotype may explain why they retained their background adaptation capabilities as well.
We have included this pomca-/- characterization in text page 16. It reads: "Unlike these mc1r knock-outs, the pomca knock-outs have relatively normal countershading (Fig. S4) as they display dark dorsums (Fig. S4A-D) and white ventrums (Fig. S4E, F). Differences in dorsal melanophores and xanthophores number were found to be statistically insignificant (Fig. S4G; p-value > 0.05), indicating that the observed pigmentation changes are indeed predominantly due to background adaptation."

Reviewer #2: (Comments to the Authors/Response to the Reviewer)

'Genetic deciphering of the antagonistic activities of the melanin-concentrating hormone and melanocortin pathways in skin pigmentation'
The authors use loss of function mutants for mchr1a/b, mchr2 and pomca, plus transgenic reporters of MCH peptide expression, to test the hypothesis that antagonistic interactions between MCH and MSH signalling control background adaptation (i.e. the rapid movement of melanosomes along the microtubular cytoskeleton to change the overall darkness of the body) in a fish genetic model, zebrafish.
The authors begin by generating a transgenic reporter of mch2 expression in zebrafish, revealing projection of MCH2-expressing hypothalamic neurons to the pituitary, and specifically their projection to regions in the vicinity of the associated vasculature. As presented the relative positions of the areas shown in each panel are difficult for the non-expert to interpret. An interpretative diagram of brain structure and the zones shown would be invaluable.

Thank you very much for the suggestion, Fig. 1 now contains two schematics (Fig. 1E' and G') describing the hypothalamic MCH projections, MCH and αMSH peptide accumulation in the pituitary regions and proximity to blood vessels.

They then used 2 distinct transgenic lines to drive heat shock-mediated overexpression of MCH1 and MCH2 respectively, compared to control non-transgenic fish that were also heat shocked. Each peptide was capable of reversing melanosome dispersion of early larval fish kept on a dark background, when expressed by heat shock.
To assess which of the candidate MCHR might mediate the MCH effect, the authors generated mchr1a mchr1b and mchr2 knockout lines, generated by CRISPR/Cas-9 or TILLING methodologies. Both 7 dpf and adult mchr2 knockouts show a pronounced constitutive dispersion of melanin, under both light and dark background conditions, as expected of the background adaptation-associated receptor. As expected, this effect was not affected by over-expression of mch1 or mch2. In contrast, neither single nor double mutants of mchr1 genes gave a pigment dispersion phenotype.
As a prelude to assessing the endogenous role of POMCA in melanosome dispersion, the authors assess tyrosinase expression in melanocytes as a method for evaluating whether melanocyte differentiation is normal. However, this requires a quantitative assessment and whole mount ISH technique they use is, at best, seim-quantitative.
The authors should supplement this panel with qRT-PCR quantitation of tyrosinase expression in the body (i.e a region excluding the eyes). Likewise, they assess pomca expression in mchr2 mutants by WISH, and by immunofluorescence, to conclude that MCHR2 knockout does not affect pomca or MSH peptide levels. Again, since they are drawing quantitative conclusions from inherently semi-quantitative techniques, more sensitive quantitative data would be useful to supplement these observations.

We agree with the reviewer that qRT-PCR is more precise way to quantify mRNA expression. However, whole mount ISH condition in zebrafish are extremely stringent and over 25 years of zebrafish research, I (Philippe Mourrain) found WISH much more reliable than qRT-PCR in term of signal to noise information and false positive frequency. Further, as discussed with the editor, Stanford campus (in)accessibility and California fires have prevented us to access our lab and core facility to perform experiments. As a consensus we modified the text to reflect the semi-quantitave signal generated by WISH.

Page 13 now reads: "*However, mchr2 mutants seems to show normal tyrosinase expression levels in melanocytes based on indistinguishable ISH expression pattern (Fig. S3A, B), suggesting that these cells develop normally in this mutant.*"

Page 14 now reads: "*In the mchr2 knock-out, we did not detect any marked variation in the expression of pomca mRNA or α-MSH small peptides based on qualitative ISH and immuno-staining (Fig. S3C-F), suggesting that the melanocortin pathway could be responsible for the skin pigmentation phenotype.*"

To test the hypothesis that melanosome dispersion depends upon POMC/a-MSH, the authors used transgenic overexpression of POMCA. Their demonstration of enhanced dispersion (Fig. S3G,H) is unclear. The larva shown in panel H seems to be deformed, with abnormal distribution of melanocytes. This makes it very difficult

to assess the dispersion of melanosomes. Closeups of individual melanocytes in morphologically unaffected regions, and quantitation of the degree of dispersion (as used throughout the rest of the paper) should be provided to justify the conclusion reached. Also, the fish shown are much younger than in the rest of the study (3 dpf) and hence close to the time when background adaptation first appears. Hence, comparison of control and transgenic fish on both white and dark backgrounds should be used to control for this.

The reviewer is correct. It is indeed complicated to assess the function of pomca/aMSH on melanosomes organization with these observations. It is because the experiment was done with the injection of a Beta-actin:pomca construct at one cell stage. This kind of experiment is transient and mosaic. The former explains why we analyzed the phenotype at 3 dpf, while the latter explains why not all melanocytes behave in the same way. As we did not have a fluorescent reporter, we were not able follow if a melanocyte received or not the transgene. We agree that this experiment is too preliminary and does not allow us to draw a definitive conclusion. Because the role of pomca/aMSH are well documented, we removed Fig. S3 G and H, and referenced previously published work to support the role of pomc/MSH in melanosomes dispersion (Sheets et al., 2007 Curr Biol., PMID:17919909 & Logan et al., 2006 Pigment Cell Res., PMID: 16704454). We modified the manuscript to reflect these modifications on page 15.

To more directly test the role for POMC, the authors generated a pomca mutant. They demonstrate both the absence of immunofluorescently-detectable aMSH and the expected failure of melanosome dispersion under light-adapted conditions. Intriguingly, an mchr2; pomca double knockout shows a 7 dpf larval phenotype indistinguishable from that of mchr2 alone, indicating that factors other than aMSH can promote melanosome dispersion. The authors observe that this changes gradually as the fish age, so that adult double mutants show the pomca knockout phenotype, indicating that another factor can substitute for MCH at this stage. These observations are intriguing and setup a series of future studies as outlined in the Discussion.
In summary, this is a conceptually simple, but convincing, well-controlled and nicely documented demonstration of the specific MCH signalling components underpinning zebrafish background adaptation.

We are extremely grateful for the reviewer's supportive comments of our study.

Minor points
1) Fig. 1 legend. Outline in panel A needs explanation.
Explanation is now provided in the legend.
2) Fig. 2D. x axis label font is too small. Enlarge so is readily legible.
Modified.
3) Fig. 3 legend. 'ROI' is not defined, although presumably 'Region of Interest'. Also, this region should be indicated/described briefly.
ROI is now spelled out and the quantified region is now defined with a box in Fig. 2 A. The same region is used in Fig. 3.
4) Fig. 5 title. As written, it does not quite make sense to me. Authors should revise.
Nice catch, thank you. Title now reads "*pomca* loss of function leads to melanosomes contraction, but is not sufficient to rescue the mchr2 mutant phenotype in larvae."

Reviewer #3: Comments to the Authors/Response to the Reviewer

In this study, Romaine and colleagues investigate the genetic mechanisms underlying skin color in teleosts (zebrafish) using transgenic models and pigmentation analysis.

These new original genetic evidence for an antagonistic role of MCH and alpha-MSH in skin pigmentation are convincing and confirm old antibody-based studies. The manuscript is well written and data are rigorously presented and analysed. I did appreciate some of the control experiment (e.g., double transgenic). The discussion of the results is appropriate. Thus, I have several comments and questions that are listed below in order of appearance in the MS.

We are really grateful for the reviewer's enthusiasm and appreciation of our genetic analysis.

The authors first map the axonal projections of MCH neurons using a transgenic line Tg(mch2:egfp) which nicely confirm original immunostaining study in salmon from the '80s. Further they show that both MCH1 and MCH 2 share pigment aggregation properties. At this point, I was wondering if MCH1 and MCH2-expressing neurons project to other targets outside the pituitary gland with some possible distinction amongst targets - this should be mentioned somewhere in the MS, at least for MCH2-expressing cells, and if not reported previously.

Thank you very much for raising this point. We previously reported the extensive innervation of periventricular regions of the adult fish brain by the MCH2 system (Berman et al., 2009 J. Comp Neurol, PMID: 19827161) including ventral telencephalon, thalamus, and posterior tuberal nucleus. We have now added this description on page 7 near the beginning of the Results section. The main commonality between both systems is the projection to the pituitary but more extensive and neuroanatomically detailed analyses will be required in future studies.

To study the receptor subtypes involved in this regulation, they then generate mchr1a, mchr1b and mchr2 KO mutants (CRISPR/Cas or TILLING) and found that only mchr2 is involved in skin pigmentation. I did appreciate the careful analysis of the double mutant [mchr1a-mchr1b] controls which did not show neither changes in pigmentation. Interestingly, control experiments using mch1 or mch2 over-expression in the mchr2 mutant confirmed the need of a functional MCHR2 to reduce melanin dispersion. This is a very convincing result.

We do appreciate your very supportive comment.

Based on previous studies showing an antagonistic action between MCH and alpha-MSH, the authors generate a KO of pomca (POMC precursor gene) fish that is not able to adapt to a black background. Yet, knock-out of pomca in the mchr2 mutant does not rescue the mchr2 and double mutant phenotype were different than the larval stage. The authors interpret these data as "... additional factors other than α-MSH can promote melanosome dispersion in the mchr2 mutant genetic background in larvae" and "...the inability of the adult mchr2 mutant to adapt to a white background is due to the predominant activity of the melanocortin pathway".
This is puzzling and I was wondering whether this could be to a low affinity cross-binding between peptide ligand (MCH/alpha-MSH - e.g., MSH onto MCHR2) and the remaining receptors. Furthermore, are the MCH and alpha-MSH peptides known to bind a single receptor (MCH or MSH receptors subtypes) with low affinity ? Could that explain this phenotype ? Homodimerization and heterodimerization are frequent amongst GPCRs - since those receptors are co-expressed in skin melanophores, could such phenomenon affect the intracellular pathway activation and, ultimately the skin pigmentation in the larval stage of these mutants?
Does a change in gene transcripts, peptide or receptor expression may explain the larval versus adult different phenotypes ? In other words, are there known changes of these across development including 'peptide-receptor' affinity ? If so, could the authors provide such experimental data ? that would really strengthen the study.

We now address these excellent points in the Discussion. We have added the section below regarding ligand cross binding and receptor dimerization on pages 23 and 24:

"Due to frequent dimerization among GPCRs (58, 59), we cannot exclude the possibility that heterodimerization of the MCR subtypes contribute to the observed discrepancy in skin phenotype. While there is no direct evidence that there is cross-binding between MCH and the melanocortin ligands with their receptor subtypes or that the ligands competitively inhibit each other's binding to their respective receptors (13), some studies suggest that MCH may have some affinity for MC5R since high concentrations of MCH can mimic the effects of α-MSH in tissues and cell cultures expressing MC5R (60). Indeed, there have been previous reports that MCH exhibits self-antagonistic, MSH-like activity at high concentrations in teleost melanophores whereby pigment dispersion is induced (61, 62) via an unknown mechanism. Although pigmentation is not mainly attributed to MC5R activity as it is with MC1R, MC5R is weakly expressed in the brain and is widely distributed in more peripheral regions like in the skin (63, 64) and is able to heterodimerize to MC1R (65). It has been reported that MC1R/MC5R heterodimerizations weakens α-MSH signaling by inhibiting cAMP accumulation and that this ligand-selective receptor complex may be able to influence color changes in teleosts (65). Thus, reduced α-MSH signaling and the self-antagonistic MCH pigment activity could be explained by competitive binding of MCH and α-MSH for the MC1R/MC5R heterodimer and this mechanism is contributing to the

different phenotypes observed in the pomca/mchr2 double knock-outs throughout development. Additionally, studies have shown that mammalian MCRs can dimerize (66-68), leading to the possibility that MC1R/MC5R dimers appear in amniotic vertebrates and can similarly affect pigmentation through balancing MCH and α-MSH activities.”

Thank you again for raising these excellent points.

Figure 2A, 3 A,B, 5 G,H all show control conditions, however, the pigmentation shape is quite different - where does that come from ? is this due to the background exposure? is there any type of correction for that across quantifications ?

We apologize if this is not clear. However, Fig. 2A and 3B show similar pigmentation shape of the melanophores in WT under a dark background while 5G is under a white background. The serrated shape is the normal contraction of melanin under a white background. The rounder shape contraction of melanin around the melanophore nuclei is a more extreme phenotype observed either in total absence of dispersing aMSH signal (Fig. 5H) or over-expression of melanin concentrating hormone signal (Fig. 2B,C).

Minors:
-many results section start with a rationale that often repeat some parts of the introduction - repetition should be avoided.
Good point, redundancies have been removed throughout the text.

-avoid the use the form "to our knowledge" multiple times.
Done.
